# TOCNF/Nisin-Stabilized Oregano Oil Pickering Emulsions Enhance Chitosan Quaternary Ammonium Salt/Propolis Films for Green Cherry Tomato Preservation

**DOI:** 10.3390/foods15183327

**Published:** 2026-09-19

**Authors:** Keying Hou, Shengsi Hu, Chenfeng Yu, Kang Tu, Xiaodong Zheng, Ye Song, Leiqing Pan

**Affiliations:** 1 College of Food Science and Technology, Nanjing Agricultural University, Nanjing 210095, China; houkeying2020@163.com (K.H.); 2024108049@stu.njau.edu.cn (S.H.); 2023108046@stu.njau.edu.cn (C.Y.); kangtu@njau.edu.cn (K.T.); 2 Jinan Fruit Research Institute, All-China Federation of Supply and Marketing Cooperatives, Jinan 250220, China; zhxd1106@163.com; 3 China Technology and Research Center for Storage and Processing of Fruit and Vegetables, Jinan 250220, China

**Keywords:** active packaging, quaternized chitosan, propolis, Pickering emulsion, oregano essential oil, green cherry tomato

## Abstract

Essential oils are difficult to disperse in hydrophilic films and may separate or volatilize during processing. We prepared chitosan quaternary ammonium salt/propolis films containing a TEMPO-oxidized cellulose nanofibril (TOCNF)/nisin-stabilized Pickering emulsion loaded with oregano essential oil. Two independent formulation variables were examined: the nisin fraction in the TOCNF/nisin particles (0–35% of particle mass) was first screened to select the emulsion stabilizer composition, whereas the selected TNO emulsion was subsequently added at 20%, 30%, 40%, or 50% (*v*/*v*) of the initial film-forming solution. At 25% nisin, encapsulation efficiency reached 88.0%, and the polydispersity index was 0.15, so this formulation was used in the films. As emulsion loading increased, the films became lighter and opacity fell from 19.75 to 4.81 A mm^−1^. HCP/TNO30 reached a tensile strength of 45.18 MPa, but elongation at break decreased from 65.35% to 28.11% across the series. Water vapor and oxygen permeability were lowest at 40% loading. HCP/TNO50 had the highest radical-scavenging and antibacterial activities. It also reduced weight loss and quality deterioration during 12 days of green cherry tomato storage. No loading optimized every property. Mechanical strength and barrier performance peaked at 30% and 40%, respectively, while active preservation continued to improve up to 50%.

## 1. Introduction

Biodegradable active packaging can slow quality loss in fresh foods while reducing the use of petroleum-derived materials [1,2]. Polysaccharides form renewable films, but their affinity for water often compromises barrier properties. Mechanical performance may also be insufficient [3]. Chitosan is attractive because it is cationic and readily forms continuous films. Quaternization broadens its useful pH range by retaining water solubility and antibacterial activity [4,5,6,7,8,9]. A practical active film, however, still requires reinforcement without sacrificing biological function.

Phenolic constituents of propolis contribute both antioxidant and antimicrobial activity [10,11,12]. Propolis extracts have also improved chitosan-based films [13,14]. Oregano essential oil creates a different formulation problem. It is antimicrobial, but its poor water dispersibility promotes separation from hydrophilic matrices [15,16,17]. Volatile constituents may be lost during film preparation or storage [18,19,20,21,22,23]. The oil must therefore be dispersed at a stable interface without disrupting the continuous film phase.

In Pickering emulsions, solid particles adsorb at the oil water interface and stabilize dispersed droplets, which can improve essential-oil dispersion and slow release [1,24,25]. TEMPO-oxidized cellulose nanofibrils (TOCNFs) are produced by TEMPO-mediated oxidation, which converts a portion of the surface hydroxyl groups of cellulose into anionic carboxylate groups. This surface chemistry promotes aqueous dispersion and enables electrostatic association with cationic nisin, while the nanofibrils can reinforce polymer networks through hydrogen bonding and physical entanglement [26,27,28,29]. Nisin also contributes antibacterial activity and has been used in active packaging [30,31,32].

Current bio-based active-packaging studies show that Pickering stabilization can facilitate the incorporation of hydrophobic essential oils into polysaccharide films, using nanocellulose, protein polysaccharide complexes, shellac nanoparticles, and related interfacial particles [1,24,33,34,35,36,37,38,39]. These studies also indicate that film performance depends strongly on the stabilizer, polymer matrix, active compound, and loading level rather than following a universal formulation rule. In the closely related work of Cai et al. [40], a shellac nanoparticle/epsilon-polylysine-stabilized antibacterial Pickering emulsion was incorporated into a quaternary-ammonium chitosan film for fruit preservation. Against this state of the art, the unresolved question is how an anionic TOCNF/cationic nisin interface functions within an HACC/propolis matrix and whether separating particle-composition screening from film-level emulsion loading reveals distinct optima for mechanical, barrier, and active-preservation performance. This formulation-specific question defines the research gap addressed in the present study.

We therefore screened the nisin fraction in TOCNF/nisin particles used to stabilize OEO. The selected emulsion was incorporated into HACC/propolis films at four volume fractions. We then related film structure to mechanical, barrier, antioxidant, and antibacterial performance and tested the films on green cherry tomatoes. Our working expectation was that intermediate loading would favor reinforcement and mass-transfer resistance, whereas higher loading would favor active preservation at the expense of flexibility.

## 2. Materials and Methods

### 2.1. Materials

Chitosan quaternary ammonium salt (HACC; purity, 98%; viscosity, 100 mPa s) was supplied by Macklin Biochemical Technology Co., Ltd. (Shanghai, China). Raw propolis was obtained from a bee farm in Zhejiang, China. The material was supplied as 100% pure bee propolis. A 1 wt% TOCNF dispersion with a carboxyl content of 1 to 2 mmol g^−1^ was purchased from Songhu Shenjian Technology Co., Ltd. (Dongguan, China). Nisin (activity, at least 1000 IU mg^−1^) and oregano essential oil (*Origanum vulgare* L.) were purchased from Macklin. Choline chloride and lactic acid were analytical-grade reagents with a stated purity of at least 98% from the same supplier. All remaining chemicals were of analytical grade unless specified otherwise.

### 2.2. Preparation of TOCNF/Nisin Particles and TNO Pickering Emulsions

#### 2.2.1. Preparation of TOCNF/Nisin Particles

Nisin was dissolved in 0.01 M hydrochloric acid to obtain a 2.5 wt% stock solution. The solution was stirred magnetically for 30 min. Meanwhile, the TOCNF dispersion was maintained at 500 rpm as the nisin solution was added dropwise. The aqueous phase contained 0.30 wt% total particles. Nisin accounted for 0%, 15%, 20%, 25%, 30%, or 35% of the particle mass. After addition, each dispersion was stirred for another 12 h to promote association between nisin and TOCNF. The nisin-free treatment contained 0.30 wt% TOCNF.

#### 2.2.2. Preparation of TNO Pickering Emulsions

OEO was added to each TOCNF/nisin dispersion at an oil-to-aqueous-phase ratio of 1:9 (*v*/*v*). The mixture was sonicated at 500 W for 5 min with an LD-FP200 ultrasonic cell disruptor (Lainde Intelligent Technology, Weifang, China) fitted with a 6 mm probe. Samples were held in an ice bath throughout sonication.

The 25% nisin formulation gave the highest encapsulation efficiency at 88.0%. It also produced the lowest PDI at 0.15. This formulation was selected for film preparation and is denoted TNO hereafter.

### 2.3. Characterization of TOCNF/Nisin Dispersions and TNO Emulsions

#### 2.3.1. Hydrodynamic Size, PDI, and Zeta Potential

Hydrodynamic size and PDI were measured by dynamic light scattering with a Zetasizer Nano ZS (Malvern Instruments, Malvern, UK). The same instrument measured zeta potential by electrophoretic light scattering. Samples were diluted 100-fold with deionized water and equilibrated for 120 s before analysis at 25 °C. All measurements were conducted with three independent replicates (*n* = 3).

#### 2.3.2. Encapsulation Efficiency

Encapsulation efficiency (EE) was determined from the non-encapsulated OEO fraction using a Varioskan Flash multimode microplate reader (Thermo Fisher Scientific, Shanghai, China). TNO samples were mixed with n-hexane to extract free OEO without deliberately disrupting the particle-stabilized interface. After centrifugation at 8000× *g* for 10 min, the upper hexane phase was collected. Free OEO was quantified at 276 nm against an OEO calibration curve. All measurements were conducted with three independent replicates (*n* = 3). EE was calculated using the following Equation (1):

(1) $$EE\;(\%)=\frac{W_{t}-W_{f}}{W_{t}}\times 100$$

where Wt is the total mass (g) of OEO added and Wf is the mass (g) of non-encapsulated OEO.

#### 2.3.3. Rheological Properties

Steady-shear behavior was measured with an MCR 302 rheometer (Anton Paar, Graz, Austria). A 50 mm parallel plate was used with a 1 mm gap. Apparent viscosity was recorded from 0.1 to 1000 s^−1^ at 25 ± 1 °C. All measurements were conducted with three independent replicates (*n* = 3).

### 2.4. DES Extraction of Propolis and Preparation of HCP/TNO Films

#### 2.4.1. Preparation of the DES-Derived Propolis Extract

Choline chloride and lactic acid were combined at a 1:2 molar ratio to prepare the deep eutectic solvent (DES). The mixture was stirred in an 80 °C water bath until it became clear and homogeneous. Raw propolis (0.5 g) was then mixed with 15 mL of DES and extracted for 2 h at 65 °C. The extract was passed through a 0.22 μm polytetrafluoroethylene syringe filter (Sartoris Lepos, Shanghai, China). The filtrate was stored at 4 °C and used within 3 days.

#### 2.4.2. Preparation of the HCP Film-Forming Solution

HACC powder (2.00 g) was dispersed in 100 mL of water and stirred at room temperature until fully dissolved, giving a 2% (*w*/*v*) solution. Liquid DES-derived propolis extract was added at 60% (*v*/*w*) relative to the dry HACC mass. Stirring continued at 500 rpm and 40 °C for 1 h to produce HCP.

#### 2.4.3. Preparation of HCP/TNO Films

TNO was blended with HCP at 0%, 20%, 30%, 40%, or 50% of the initial HCP-solution volume. We refer to these formulations as HCP, HCP/TNO20, HCP/TNO30, HCP/TNO40, and HCP/TNO50. Mixing continued at 500 rpm and 40 °C for 1 h. A 30 mL aliquot of each mixture was poured onto a circular plate 14 cm in diameter. After drying at 35 °C for 24 h, the films were removed from the plates and conditioned at 25 °C and 75% RH for approximately 48 h before analysis.

### 2.5. Film Characterization

#### 2.5.1. Appearance

For visual comparison of transparency, each film was placed over the same printed reference pattern and photographed under fixed lighting with a Lumix S5II digital camera (Panasonic, Osaka, Japan). Photographs were compared for visible color and transparency.

#### 2.5.2. Color and Opacity

We recorded L*, a*, and b* coordinates with a WR-10 colorimeter (FRU, Shenzhen, China). The following equation was used to obtain total color difference (ΔE). Reference coordinates were L_0_* = 99.49, a_0_* = −0.07, and b_0_* = −0.16. ΔE was calculated using the following Equation (2):

(2) $$\Delta E=\sqrt{{\left(L^{*}-L_{0}\right)}^{2}+{\left(a^{*}-a_{0}\right)}^{2}+{\left(b^{*}-b_{0}\right)}^{2}}$$

Opacity was determined with an LC-V1200E UV-visible spectrophotometer (Lichen, Shanghai, China). A 1 × 4 cm film strip was inserted into an empty glass cuvette. A second empty cuvette provided the reference. Absorbance was read at 600 nm. Opacity was calculated using the following Equation (3):

(3) $$Opacity=\frac{A_{600}}{X}$$

In this expression, A600 denotes absorbance at 600 nm, and X denotes film thickness in millimeters.

#### 2.5.3. Thickness

A DL321025S digital micrometer (Deli Group Co., Ltd., Ningbo, China) was used to record film thickness. Five locations were measured on every specimen, and their average entered the subsequent calculations.

#### 2.5.4. Mechanical Properties

TS and EB were measured with a TMS-PRO texture analyzer (Food Technology Corporation, Chicago, IL, USA). The specimens were film strips measuring 90 × 10 mm. Initial grip separation was 40 mm, and the crosshead traveled at 100 mm min^−1^. Three strips from each formulation were tested. TS was calculated using the following Equation (4):

(4) $$TS=\frac{F}{W\times H}$$

EB was calculated using the following Equation (5):

(5) $$EB=\left[\frac{L_{b}-L_{0}}{L_{0}}\right]\times 100\%$$

In these equations, F is the maximum tensile force in newtons. W and H are specimen width and thickness in millimeters. L0 denotes the initial grip separation, while Lb denotes the grip separation at break. Both lengths are expressed in millimeters.

#### 2.5.5. Water Vapor Permeability

Water vapor permeability (WVP) was determined by the gravimetric cup method according to ASTM E96/E96M-24a [41]. Circular specimens were sealed over cups containing anhydrous calcium chloride. Each cup had a 60 mm inner diameter and an exposed area of 28.27 cm^2^. The assembled cups were held at 25 ± 1 °C and 75 ± 2% RH. Their mass was recorded every 2 h for 24 h. Water vapor transmission rate was obtained from the linear slope of mass gain against time. Three independently prepared films were tested per formulation. WVP was calculated using the following Equation (6):

(6) $$WVP=\frac{\Delta m\times L}{A\times t\times \Delta P}$$

In the equation, Δm/t is the mass-gain slope in g h^−1^, and L is mean film thickness in millimeters. A represents the exposed area in square meters. ΔP is the water vapor partial-pressure difference across the film in kilopascals.

#### 2.5.6. Oxygen Permeability

OP was determined according to ASTM D3985-24 [42] using an OX2/230 Oxygen Permeation Analyzer (MOCON Inc., Minneapolis, MN, USA). Circular specimens exposed an effective area of 5 cm^2^. High-purity oxygen and nitrogen were supplied separately, each at 10 mL min^−1^. Testing began after equilibration at 23 ± 0.5 °C and 50 ± 2% RH. Three independently prepared films represented each formulation. The analyzer derived OP from the steady-state oxygen transmission rate and reported it as cm^3^ m^−2^ h^−1^ kPa^−1^. OP was calculated using the following Equation (7):

(7) $$OP=\frac{Q}{A\times t\times \Delta P}$$

In this expression, Q denotes oxygen throughput in cubic centimeters, whereas A denotes exposed area in square meters. Time t is expressed in hours, and ΔP is the oxygen partial-pressure difference in kilopascals.

#### 2.5.7. Water Contact Angle

Water contact angle was measured with an OCA 20 AMP optical contact-angle analyzer (Biolin Scientific AB, Gothenburg, Sweden). Before measurement, each film specimen was placed with the air-exposed surface facing upward and fixed flat onto a clean glass slide using thin double-sided adhesive tape applied to the reverse side and along the edges of the film. Care was taken to ensure that the adhesive tape did not contact the central measurement area. The film was gently flattened to eliminate wrinkles, air gaps, or local curvature and to maintain a horizontal test surface. Subsequently, a 20 μL droplet of deionized water was carefully deposited onto the film surface, and the contact angle was recorded immediately after droplet deposition. Three measurements were obtained at different positions for each formulation.

#### 2.5.8. Morphological and Structural Characterization

ATR-FTIR spectra were recorded with a Nicolet iS5 spectrometer (Thermo Fisher Scientific, Waltham, MA, USA). Each spectrum covered 4000 to 500 cm^−1^ and comprised 64 scans at a resolution of 4 cm^−1^.

Film surfaces and cross sections were examined with a GeminiSEM 300 field-emission scanning electron microscope (ZEISS, Oberkochen, Germany). Gold-coated specimens were imaged at 10 kV. Surface images were acquired at ×10,000 magnification, whereas cross sections were recorded at ×20,000.

X-ray diffraction patterns were recorded with an Ultima IV diffractometer (Rigaku, Tokyo, Japan) using Cu Kα radiation at 40 kV and 50 mA. Data were collected from 2θ = 5° to 60° at 2° min^−1^.

### 2.6. Antioxidant and Antibacterial Assays

#### 2.6.1. Antioxidant Activity

Film extracts were prepared by stirring 0.5 g of film in 10 mL of 95% methanol for 3 h in darkness. For the DPPH assay, 0.5 mL of extract was combined with 1.0 mL of 0.1 mM methanolic DPPH. The mixture remained in darkness for 30 min at room temperature before measurement at 517 nm. For the ABTS assay, 50 μL of extract was added to 3.0 mL of methanolic working solution. Its initial absorbance was 0.700 ± 0.020 at 734 nm. Absorbance was recorded at 734 nm after 6 min in darkness at room temperature. Each assay was performed in triplicate. DPPH was calculated using the following Equation (8):

(8) $$DPPH\;scavenging\;(\%)=\frac{A_{0}-A_{s}}{A_{0}}\times 100$$

where A_0_ is the blank absorbance and As is the sample absorbance.

ABTS radical-scavenging activity was calculated using the following Equation (9):

(9) $$ABTS\;scavenging\;(\%)=\frac{A_{0}-A_{s}}{A_{0}}\times 100$$

where A_0_ is the blank absorbance and As is the sample absorbance.

#### 2.6.2. Antibacterial Activity

Antibacterial activity was assessed against Escherichia coli ATCC 25922 and Staphylococcus aureus ATCC 6538. Each strain was cultured in nutrient broth at 37 °C and 180 rpm for 18 h. Suspensions were adjusted to approximately 1 × 10^7^ CFU mL^−1^. Sterilized film specimens measuring 20 × 20 mm and weighing approximately 50 mg were immersed in 10 mL of bacterial suspension. Samples were incubated at 37 °C and 120 rpm for 24 h. Serial dilutions were then spread onto nutrient agar plates. Viable colonies were counted after incubation at 37 °C for 24 h. PE film served as the control. Bacteriostatic rate was calculated using the following Equation (10):

(10) $$Antibacterial\;rate\;(\%)=\frac{{CFU}_{control}-{CFU}_{sample}}{{CFU}_{control}}\times 100$$

CFU_control_ is the viable count obtained for the PE control. CFU_sample_ is the corresponding count after film treatment.

### 2.7. Green Cherry Tomato Preservation Test

Green cherry tomatoes (*Solanum lycopersicum* var. cerasiforme) at the mature-green stage were obtained from a commercial supplier in Jiangsu, China. Before treatment assignment, each fruit was inspected. Tomatoes with physical damage, disease, insect injury, cracking, decay, or other visible defect were discarded. The remaining fruits were randomly assigned to film treatments and stored for 12 days at 25 ± 2 °C and 80 ± 5% RH. Tomatoes packaged in PE provided the reference. Appearance and weight loss were assessed on days 0, 3, 6, 9, and 12. Firmness, total soluble solids (TSS), and titratable acidity (TA) were determined at the same time points. For firmness, a TMS-PRO texture analyzer fitted with a 2 mm cylindrical stainless-steel probe advanced 5 mm into the fruit at 1.0 mm s^−1^. Force was recorded in newtons. TSS was measured in filtered tomato juice with a handheld refractometer (ATAGO, Tokyo, Japan) and reported as °Brix. For TA, 10.0 g of homogenate was dispersed in 40 mL of distilled water. A 10.0 mL aliquot was titrated with 0.100 mol L^−1^ NaOH to pH 8.1 ± 0.1.

Green cherry tomatoes were placed in plastic boxes (7 × 7 × 4.5 cm). For the film-treated groups, the corresponding film was positioned over the open top of each box and secured around the rim with a rubber band to maintain its position throughout storage.

Weight loss and TA were calculated using the following Equations (11) and (12):

(11) $$Weight\;Loss\;Rate\;(\%)=\frac{M_{1}-M_{0}}{M_{0}}\times 100\%$$

where M_0_ is tomato mass (g) before storage and M_1_ is tomato mass (g) at the specified sampling time.

(12) $$TA\;(\%)=\frac{C\left(V-V_{0}\right)fV_{1}}{V_{2}m}\times 100$$

where C is the NaOH concentration, V and V_0_ are the sample and blank titration volumes in milliliters, V_1_ is the total volume of the sample, V_2_ is the aliquot volume used for titration, f is 0.064 g citric acid per milliliter of 0.1 mol L^−1^ NaOH. m is the mass of tomato homogenate.

### 2.8. Statistical Analysis

Unless stated otherwise, data are mean ± standard deviation from three independent replicates. One-way analysis of variance assessed single-time-point measurements. Storage data were analyzed by two-way analysis of variance with treatment and time as fixed factors. Separate tomatoes were sampled at each time point. Three independent packages were prepared for every treatment and treated as independent storage replicates. Duncan’s multiple-range test was applied in SPSS Statistics 27 (IBM, Armonk, NY, USA), with *p* < 0.05 considered significant. Prior to ANOVA/Duncan’s test, the assumptions of normality and homogeneity of variances were evaluated.

## 3. Results and Discussion

### 3.1. Selection and Physicochemical Properties of the TNO Pickering Emulsion

**Figure 1:**
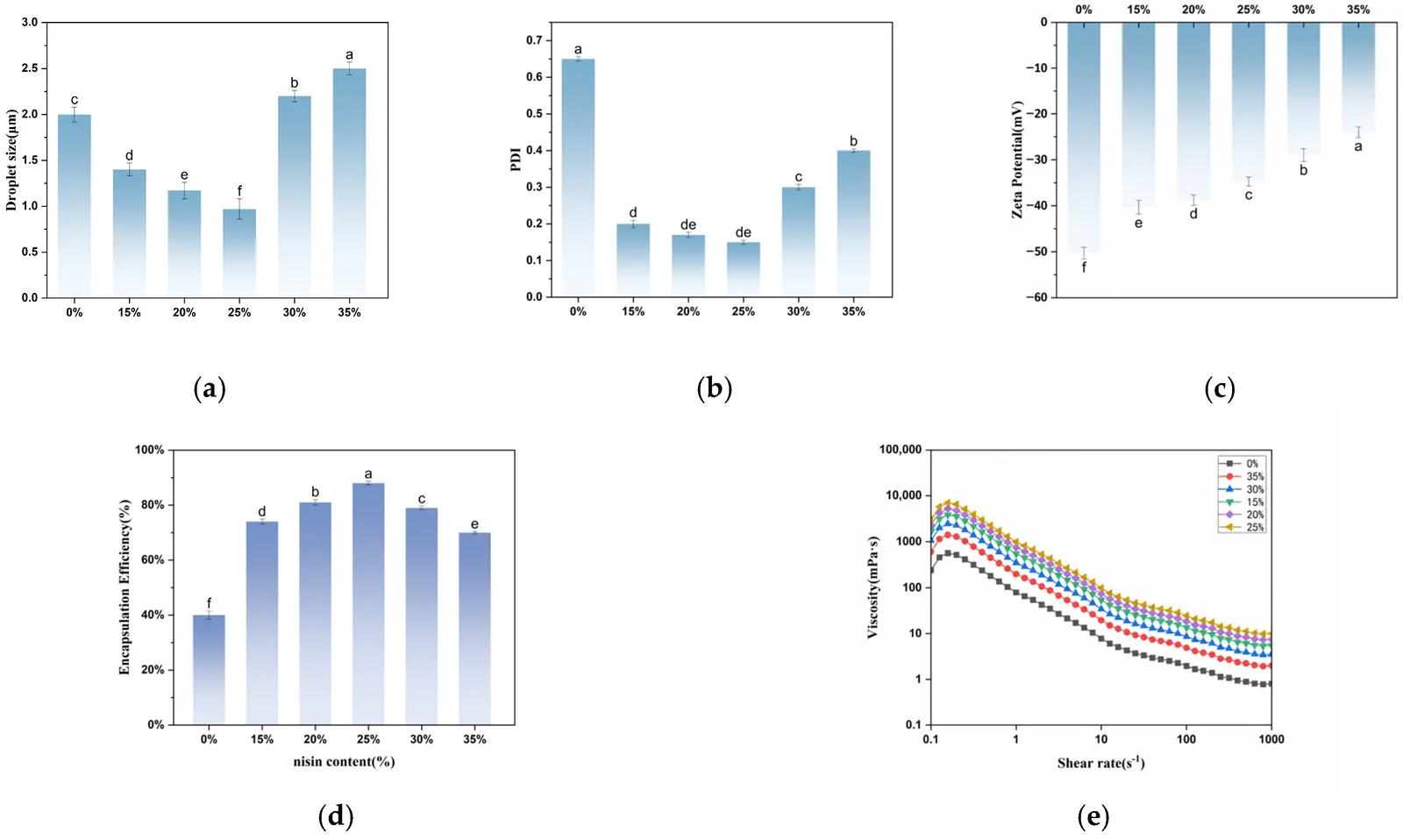
Optimization and characterization of the TNO Pickering emulsion. From left to right and top to bottom, the panels show droplet size (**a**), PDI (**b**), zeta potential (**c**), OEO encapsulation efficiency (**d**), and apparent viscosity (**e**) across nisin fractions from 0% to 35%. Values are mean ± SD (*n* = 3). Different lowercase letters indicate significant differences at *p* < 0.05.

Nisin changed the droplet-size distribution and zeta potential of the TNO emulsions (Figure 1). Without nisin, mean droplet size was about 2.0 μm. It fell to nearly 1.0 μm at 25% nisin before increasing at higher fractions. PDI likewise reached its minimum of 0.15 at 25%. Zeta potential moved toward zero as more nisin was added, shifting from approximately −50 mV at 0% to −24 mV at 35%. This trend is compatible with nisin association at the TOCNF-stabilized interface, although the measurements do not resolve how binding occurred [28,29,38,43].

Encapsulation efficiency increased from 40.0% without nisin to 88.0% at a 25% nisin fraction, before falling to 79.0% and 70.0% at 30% and 35%, respectively. Every emulsion was shear-thinning, and apparent viscosity generally rose with nisin content. The 25% formulation combined the smallest droplets, the narrowest size distribution, and the highest encapsulation efficiency. It was therefore carried forward for film preparation.

The nisin fraction varied during emulsion selection, and the TNO volume fraction varied during film formulation, and are independent experimental variables.

### 3.2. Film Appearance, Color, and Opacity

**Figure 2:**
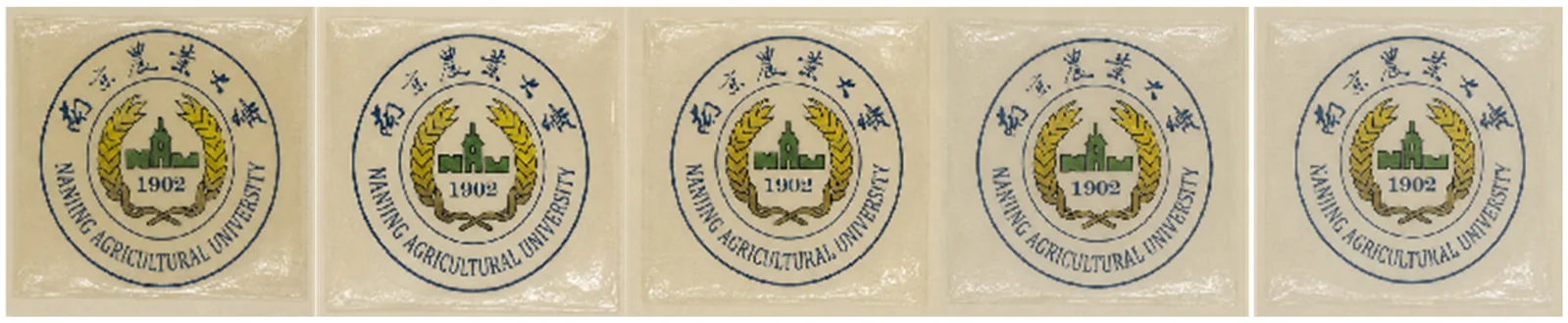
Visual transparency of HCP and HCP/TNO films placed over the same printed reference pattern. From left to right, samples are HCP, HCP/TNO20, HCP/TNO30, HCP/TNO40, and HCP/TNO50. The increasing visibility of the pattern provides a qualitative comparison of transparency and is consistent with the increase in L* and the decreases in b* and opacity reported in Table 1.

TNO changed the films both visually and instrumentally (Figure 2; Table 1). L* rose from 85.62 in HCP to 97.22 in HCP/TNO50, whereas b* fell from 15.37 to 1.36. Accordingly, the films looked lighter and less yellow [25,44,45,46]. Using the reference coordinates in Section 2.5.2, ΔE decreased from 20.82 to 2.92.

Opacity fell steadily from 19.75 A mm^−1^ in HCP to 4.81 A mm^−1^ in HCP/TNO50 (Table 1), indicating less attenuation at 600 nm. This change could arise from a more uniform optical dispersion [47,48]. Dilution of the colored propolis phase is also possible, and the current measurements do not distinguish the two explanations.

### 3.3. Microstructure, Molecular Interactions, and Crystallinity

**Figure 3:**
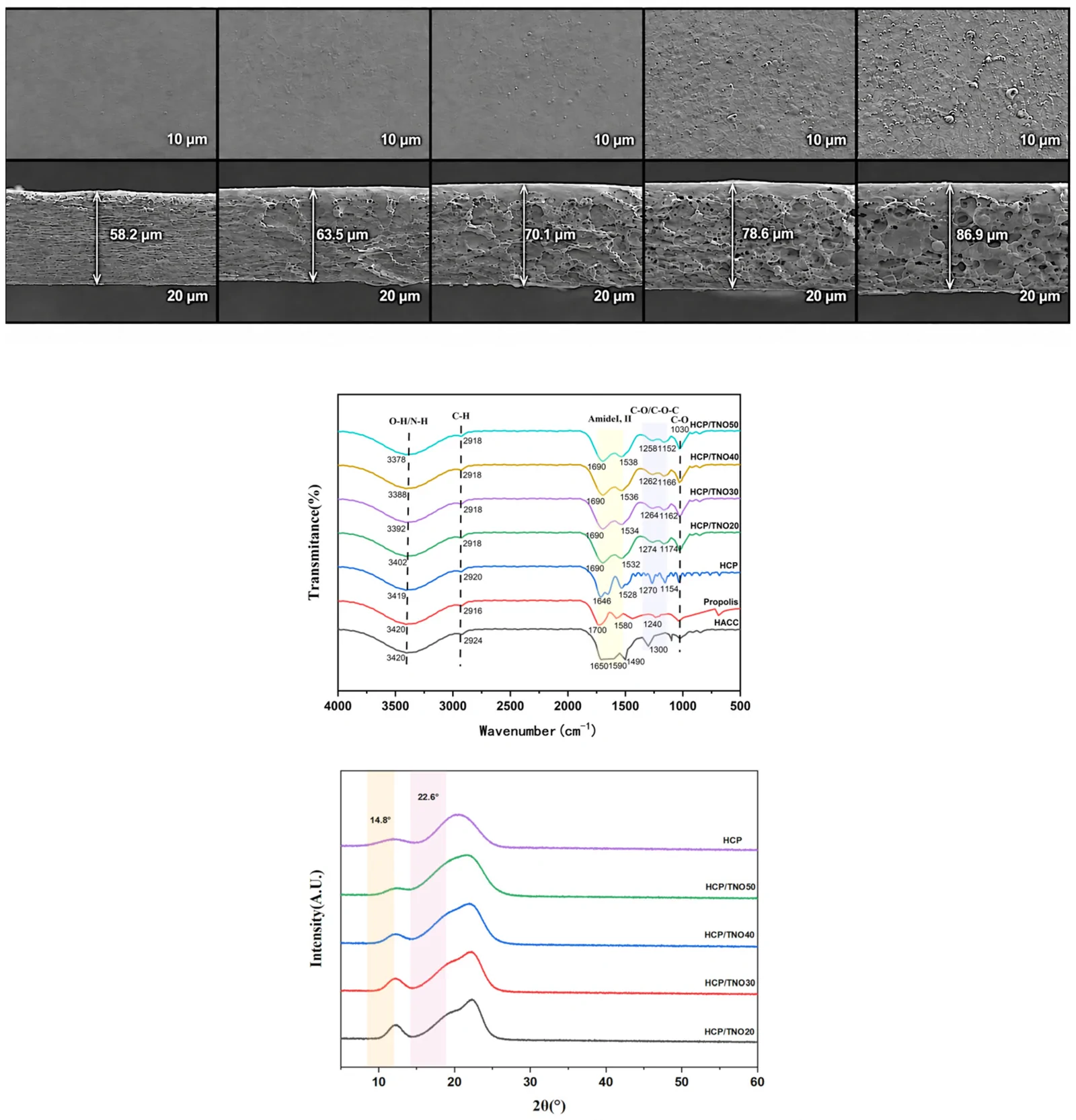
Morphological and structural characterization of HCP and HCP/TNO films. In the SEM block, columns from left to right correspond to HCP, HCP/TNO20, HCP/TNO30, HCP/TNO40, and HCP/TNO50; the upper and lower rows show surface (×10,000) and cross-sectional (×20,000) micrographs, respectively. Thickness values marked on the cross-sectional panels are local measurements at the indicated positions in the displayed fields and are not formulation means. The lower plots show ATR-FTIR spectra (4000–500 cm^−1^) and XRD patterns (2theta = 5–60 degrees). Values are mean ± SD (*n* = 3).

The HCP surface was smooth, but roughness increased progressively after TNO incorporation (Figure 3). The cross sections changed from a compact, layered structure to a more porous and heterogeneous one. Thickness labels in the micrographs increased from 58.2 μm for HCP to 86.9 μm for HCP/TNO50 [24,29,49]. The dispersed phase was therefore incorporated without producing a uniformly denser film at high loading. The greater porosity at 50% TNO may account for the partial rebound in permeability.

The thickness values annotated in the cross-sectional SEM panels and those in Table 2 were obtained by different measurement approaches. The SEM annotations describe local cross-sectional thicknesses at the indicated positions in the displayed fields, whereas Table 2 reports micrometer-based specimen thickness averaged across five locations. Because the SEM measurement samples a local cross section while the micrometer result averages a broader set of positions, the two sets of values need not coincide; Table 2 was used for the quantitative film-property calculations.

TNO loading also altered the ATR-FTIR spectra (Figure 3). The broad O-H/N-H band at 3419 cm^−1^ in HCP shifted to 3402, 3392, 3388, and 3378 cm^−1^ as loading rose from 20% to 50%. Changes were also evident in the carbonyl/amide and C-O/C-O-C regions. The shifts are compatible with non-covalent interactions in the composite matrix [22,50]. Hydrogen bonding and electrostatic association remain plausible explanations, although the spectra provide no evidence of covalent grafting.

XRD patterns contained broad features near 2θ 14.8° and 22.6° (Figure 3). Both features persisted after TNO was added, but their relative intensities varied. The emulsion modified short-range order without creating a new dominant crystalline phase [4,12].

### 3.4. Mechanical Properties and Thickness

TS varied nonlinearly with TNO loading (Table 2). Starting from 15.93 MPa in HCP, it increased to 30.42 MPa at 20% and reached 45.18 MPa in HCP/TNO30. The latter value was approximately 184% higher than that in HCP. TS then declined to 33.66 MPa at 40% and 20.07 MPa at 50% [2,24,48]. Within the tested range, reinforcement of the polymer-particle network was greatest at 30% loading.

The loading series showed the opposite pattern for extensibility. EAB fell from 65.35% in HCP to 28.11% in HCP/TNO50. At the same time, film thickness increased from 0.050 mm in HCP to 0.069 to 0.074 mm in the TNO-containing formulations. Thickness cannot account for the TS increase because tensile stress is normalized by cross-sectional area. The results instead point to interfacial reinforcement accompanied by lower chain mobility [21,51,52].

### 3.5. Barrier Properties and Surface Wettability

**Figure 4:**
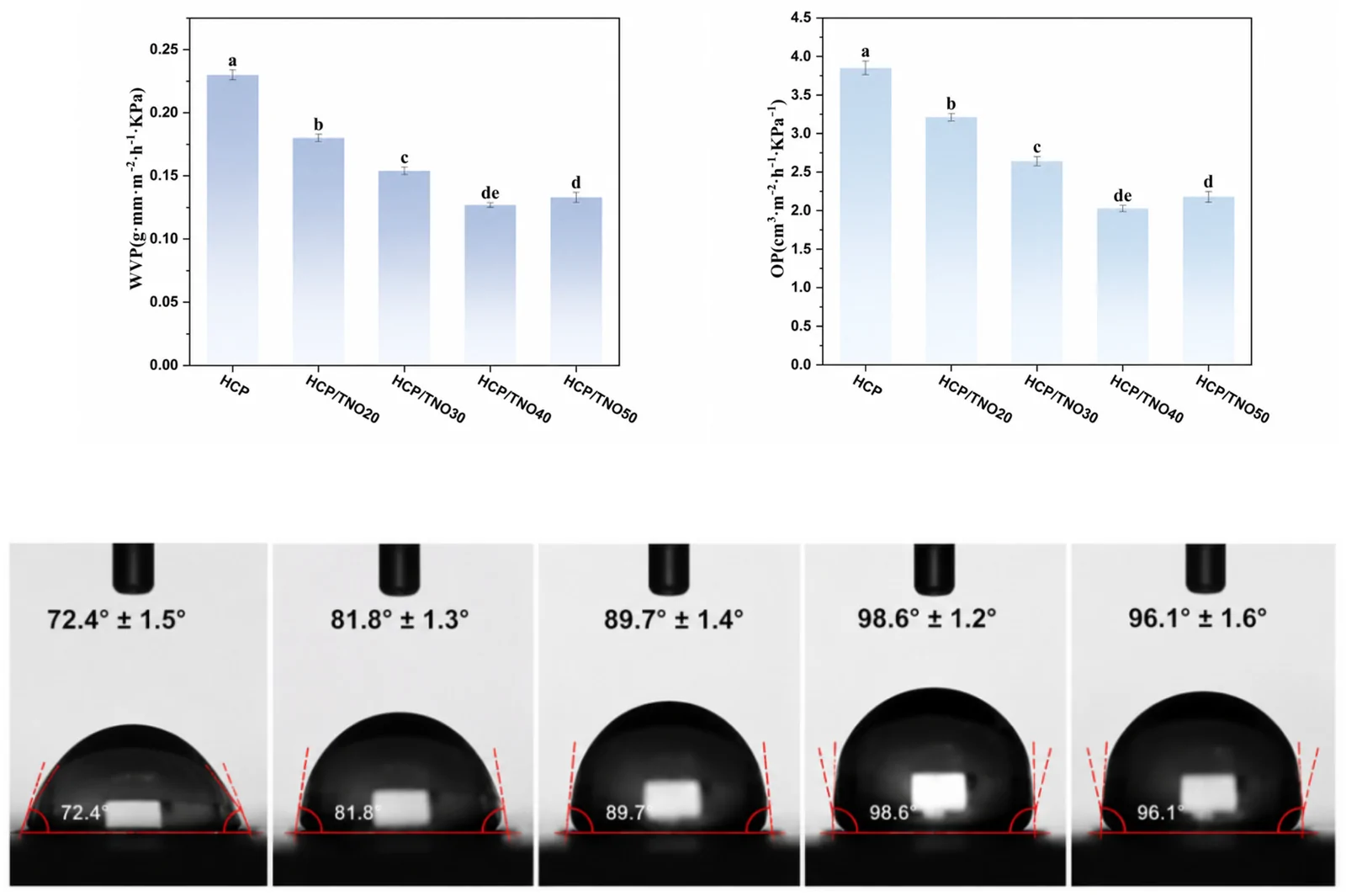
Barrier properties and surface wettability of HCP and HCP/TNO films. The panels show water vapor permeability, oxygen permeability, and water contact angle. Values are mean ± SD (*n* = 3). Different lowercase letters indicate significant differences at *p* < 0.05.

Barrier performance improved as TNO loading increased to 40% (Figure 4). WVP fell from 0.230 g·mm·m^−2^·h^−1^·kPa in HCP to 0.127 g·mm·m^−2^·h^−1^·kPa in HCP/TNO40. At 50% loading, it rose slightly to 0.133 g·mm·m^−2^·h^−1^·kPa. OP followed the same pattern. Values decreased from 3.85 in HCP to 2.03 in HCP/TNO40, then increased to 2.18 in HCP/TNO50. OP was expressed as cm^3^·m^−2^·h^−1^·kPa^−1^. Hydrophobic oil domains may lengthen diffusion paths at intermediate loading [19,22,34,48]. The heterogeneous cross section seen at 50% offers a plausible explanation for the subsequent rebound.

Water contact angle rose from 72.4° in HCP to 98.6° in HCP/TNO40 (Figure 4). It then declined slightly to 96.1° at 50% loading. TNO therefore reduced surface wettability [33,35,38]. Surface enrichment of hydrophobic OEO could contribute, but direct surface-composition measurements would be needed to test that interpretation.

### 3.6. Antioxidant Activity

**Figure 5:**
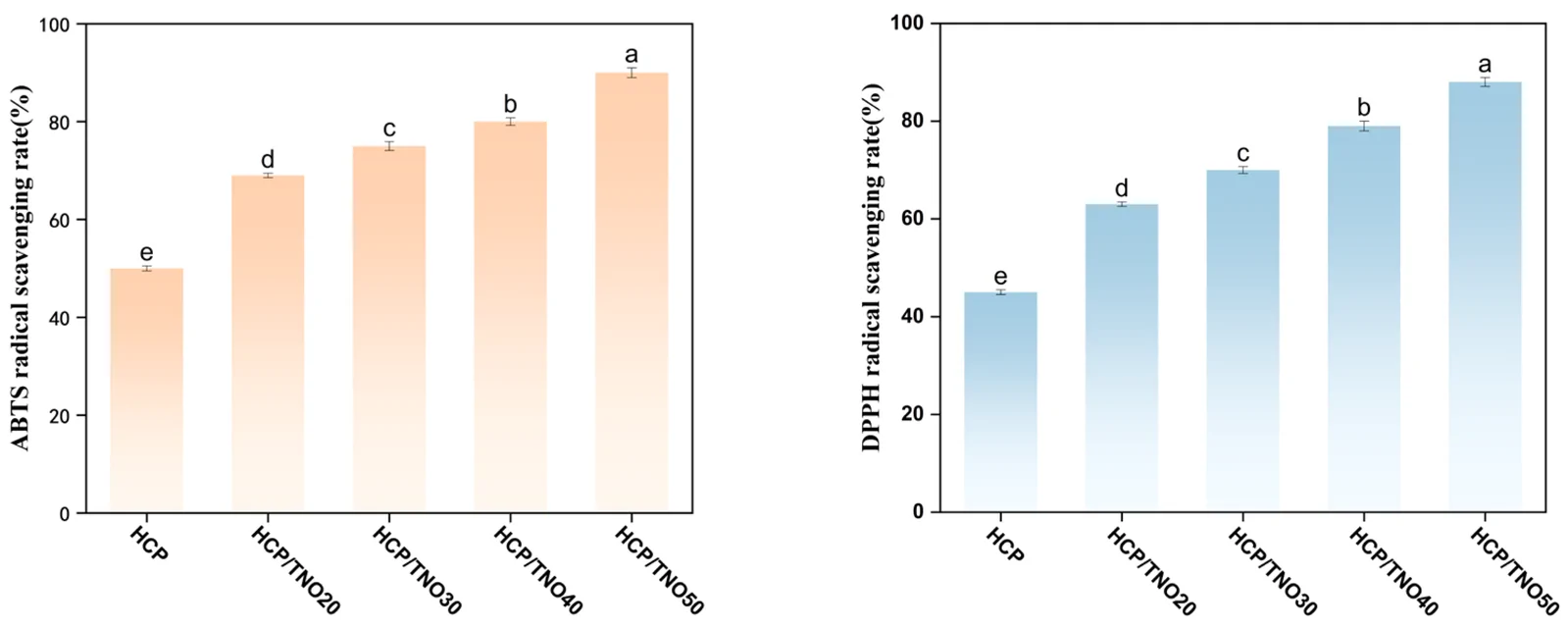
ABTS and DPPH radical-scavenging activities of HCP and HCP/TNO films. Values are mean ± SD (*n* = 3). Different lowercase letters indicate significant differences at *p* < 0.05.

Radical-scavenging activity rose across the TNO loading series (Figure 5). ABTS activity increased from 50% in HCP to 90% in HCP/TNO50, while DPPH activity rose from 45% to 88%. The emulsion contributed additional radical-scavenging capacity beyond that of the HACC/propolis matrix [35,48,49,51].

The DPPH and ABTS assays represent the in vitro radical-scavenging capacity of methanol-extractable compounds from the films. Because matched single-component controls were not included, the individual contributions of OEO, nisin, and TOCNFs cannot be distinguished. Moreover, methanolic extraction does not directly reflect the release or availability of antioxidant compounds under actual food-packaging conditions. Therefore, these values should be interpreted together with the tomato preservation results as complementary indicators of film functionality rather than as direct evidence of oxidative control inside the package. Further studies using aqueous extracts, food simulants, and direct oxidation measurements in packaged foods are required to determine the antioxidant activity available under conditions more representative of practical packaging applications.

### 3.7. Antibacterial Activity

**Figure 6:**
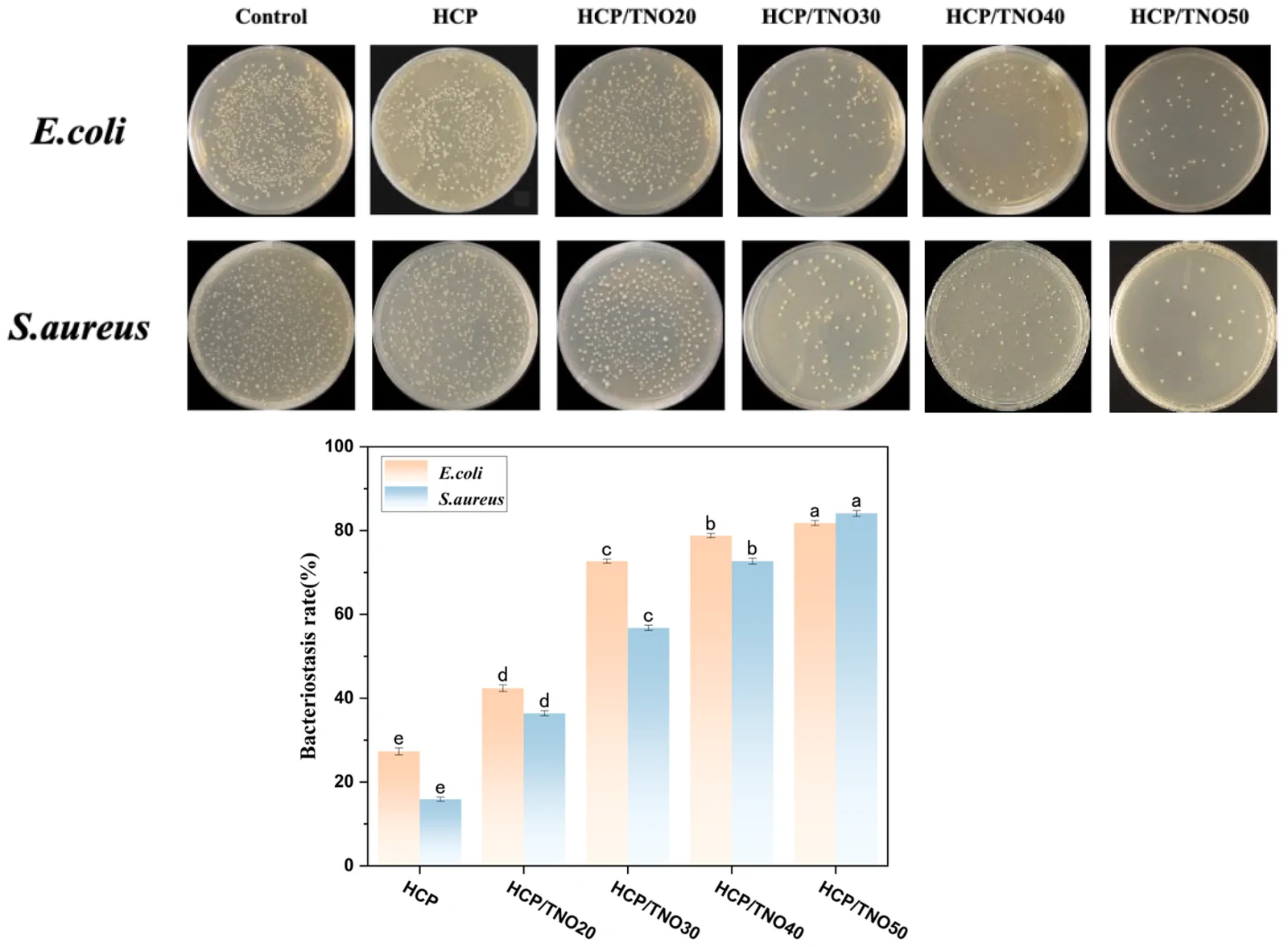
Antibacterial activity of HCP and HCP/TNO films. The figure presents representative viable-count plates and bacteriostatic rates for Escherichia coli and Staphylococcus aureus. Values are mean ± SD (*n* = 3). Different lowercase letters indicate significant differences at *p* < 0.05.

TNO-containing films inhibited both test organisms more strongly than HCP (Figure 6). Against E. coli, the bacteriostatic rate increased from about 27% in HCP to more than 80% in HCP/TNO50. S. aureus showed a similar loading response. Its rate rose from 15.9% in HCP to 84.1% at 50% TNO [29,33,43,53].

The loading response is compatible with the reported antibacterial functions of quaternized chitosan, nisin, and OEO [4,5,6,7,20,30,31,32,52,54]. Synergy cannot be inferred, however, because the experiment did not include films with only nisin, only OEO, or non-encapsulated OEO. The data support stronger combined activity, but not a synergistic mechanism.

### 3.8. Preservation Performance During Green Cherry Tomato Storage

**Figure 7:**
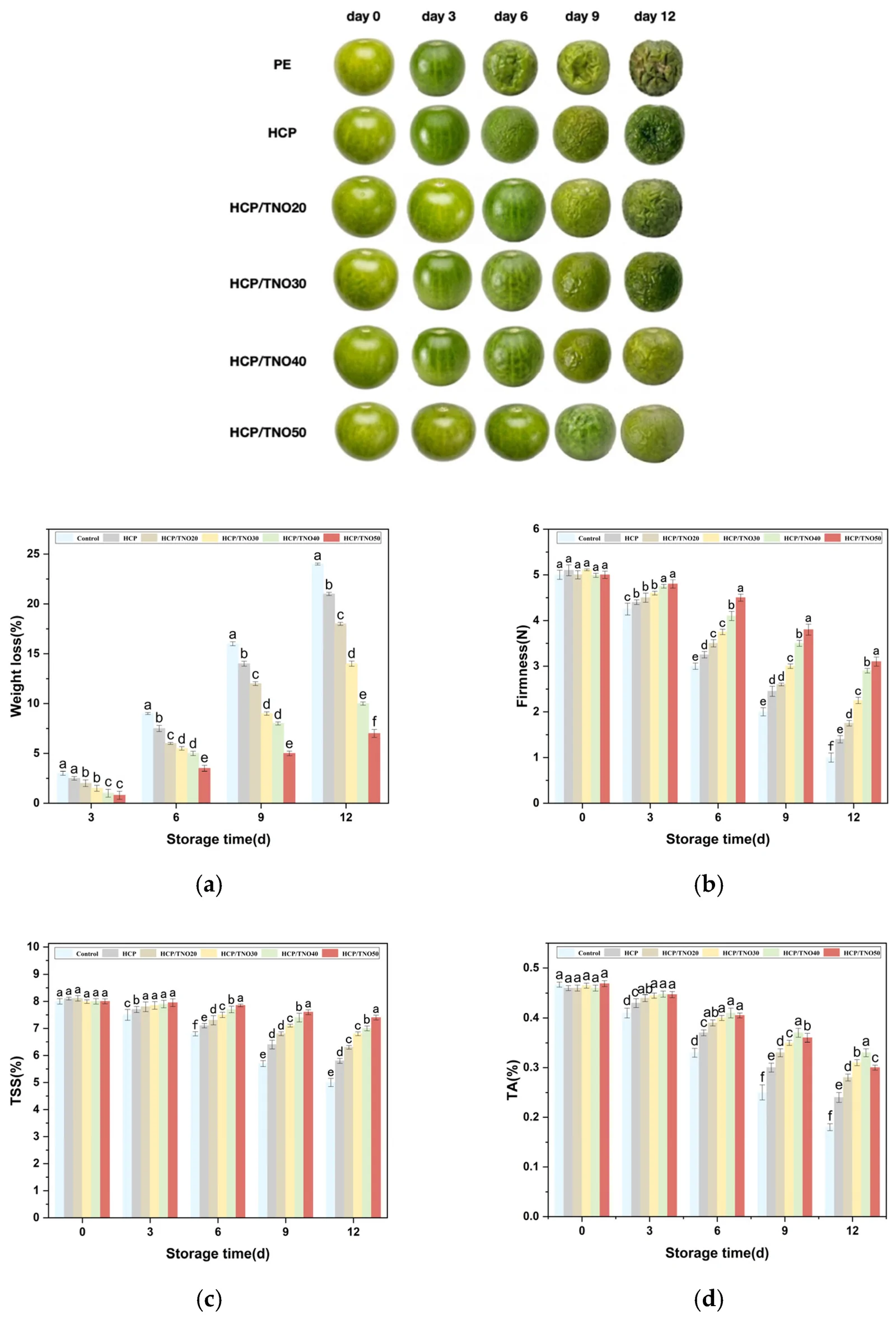
Green cherry tomatoes packaged with PE, HCP, or HCP/TNO films during 12 days of storage. From top to bottom, the panels show appearance, weight loss (**a**), firmness (**b**), total soluble solids (**c**), and titratable acidity (**d**). Values are mean ± SD (*n* = 3). Different lowercase letters indicate significant differences among treatments at each sampling time (*p* < 0.05).

Weight loss increased in every treatment during storage and was greatest in the PE and HCP packages (Figure 7). TNO-containing films reduced the increase, with HCP/TNO50 giving the lowest value on day 12. This pattern agrees with the lower WVP and surface wettability of the TNO films [16,55,56,57,58]. Its magnitude cannot be assigned to the barrier alone because package geometry and sealing conditions were not reported.

Firmness decreased over 12 days, although HCP/TNO40 and HCP/TNO50 retained higher values than PE and HCP. Higher TNO loadings also moderated the changes in TSS and TA. The films thus delayed quality deterioration, but the experiment cannot separate barrier effects from the action of released compounds [22,49,50,59,60]. Preservation favored 40% to 50% TNO, whereas tensile strength peaked at 30%, so the appropriate formulation depends on the packaging objective.

## 4. Conclusions

Screening identified the 25% nisin emulsion as the best-performing formulation within the tested set, with 88.0% encapsulation efficiency and a PDI of 0.15. Its effects after incorporation into HACC/propolis films were strongly dependent on loading. HCP/TNO30 gave the highest tensile strength, whereas permeability was lowest in HCP/TNO40. At 50% loading, radical-scavenging and antibacterial activities were greatest, and tomato quality was retained most effectively. This higher loading also reduced flexibility and increased cross-sectional porosity. The appropriate formulation therefore depends on the intended packaging function. Before food application, performance should be checked in additional independent storage batches, and sensory effects should be assessed. Migration, release behavior, and complete processing conditions also require evaluation and reporting.

Translation beyond laboratory casting requires evaluation at three levels. First, scalability and economic viability should be established through continuous mixing, coating, and drying trials, together with batch-consistency, energy-demand, raw-material-cost, and commercial-package comparisons. Second, because oregano essential oil and propolis may alter product aroma or flavor, sensory thresholds and consumer acceptance must be assessed; no organoleptic evaluation was performed in the present study. Third, food-contact use will require migration and release testing, dietary-exposure assessment, and verification that the ingredients and finished film comply with the regulations of the intended market. The present functional and storage data therefore demonstrate laboratory-scale potential but do not by themselves establish industrial feasibility, sensory acceptability, regulatory approval, or food-contact safety.

## Figures and Tables

**Table 1:** Color coordinates and opacity of HCP and HCP/TNO films.

Samples	L*	a*	b*	ΔE	Opacity (A mm^−1^)
HCP	85.62 ± 0.87 e	−0.34 ± 0.03 a	15.37 ± 0.17 a	20.82 ± 0.27 a	19.75 ± 1.1 a
HCP/TNO20	90.03 ± 0.51 d	−0.57 ± 0.62 b	9.74 ± 0.36 b	13.70 ± 0.42 b	10.35 ± 0.17 b
HCP/TNO30	93.12 ± 1.09 c	−0.96 ± 0.96 c	7.68 ± 0.12 c	10.14 ± 0.99 c	8.11 ± 0.08 c
HCP/TNO40	95.50 ± 0.30 b	−0.93 ± 0.39 c	4.15 ± 0.88 d	5.94 ± 0.03 d	5.49 ± 0.32 d
HCP/TNO50	97.22 ± 0.48 a	−1.11 ± 0.29 d	1.36 ± 1.24 e	2.92 ± 0.89 e	4.81 ± 0.26 e

Values are mean ± SD (*n* = 3). Different lowercase letters within a column indicate significant differences (*p* < 0.05).

**Table 2:** Mechanical properties and thickness of HCP and HCP/TNO films.

Samples	TS (MPa)	EAB (%)	Thickness (mm)
HCP	15.93 ± 0.33 e	65.35 ± 0.3 a	0.05 ± 0.0015 c
HCP/TNO20	30.42 ± 0.51 c	49.61 ± 0.45 b	0.069 ± 0.001 ab
HCP/TNO30	45.18 ± 0.47 a	38.80 ± 1.51 c	0.071 ± 0.0038 a
HCP/TNO40	33.66 ± 0.39 b	30.72 ± 0.22 d	0.073 ± 0.0018 a
HCP/TNO50	20.07 ± 0.26 d	28.11 ± 0.31 e	0.074 ± 0.0029 a

Values are mean ± SD (*n* = 3). Different lowercase letters within a column indicate significant differences (*p* < 0.05).

## Data Availability

The original contributions presented in this study are included in the article. Further inquiries can be directed to the corresponding authors.

## References

[1] Pandita G., de Souza C.K., Gonçalves M.J., Jasińska J.M., Jamróz E., Roy S. (2024). Recent progress on Pickering emulsion stabilized essential oil added biopolymer-based film for food packaging applications: A review. Int. J. Biol. Macromol..

[2] Wang H., Xu Z., Jin X., Hu J., Tao Y., Lu J., Xia X., Tan M., Du J., Wang H. (2025). Structurally robust chitosan-based active packaging film by Pickering emulsion containing tree essential oil for pork preservation. Food Chem..

[3] Siripatrawan U., Kaewklin P. (2018). Fabrication and characterization of chitosan-titanium dioxide nanocomposite film as ethylene scavenging and antimicrobial active food packaging. Food Hydrocoll..

[4] Hu D., Wang L. (2016). Fabrication of antibacterial blend film from poly (vinyl alcohol) and quaternized chitosan for packaging. Mater. Res. Bull..

[5] Kim Y.H., Kim H.J., Yoon K.S., Rhim J.W. (2023). Cellulose nanofiber/deacetylated quaternary chitosan composite packaging film for growth inhibition of Listeria monocytogenes in raw salmon. Food Packag. Shelf Life.

[6] Min T., Zhu Z., Sun X., Yuan Z., Zha J., Wen Y. (2020). Highly efficient antifogging and antibacterial food packaging film fabricated by novel quaternary ammonium chitosan composite. Food Chem..

[7] Sun B., Xu F., Chen D., Liu J. (2025). Quaternary ammonium chitosan-based active packaging films incorporated with dialdehyde guar gum-proanthocyanidins conjugates: Characterization and application in the edible coating of pork. Food Hydrocoll..

[8] Zhu X., Yang P., Zeng Y., Chen L., Guo X., Kang T. (2025). An antimicrobial packaging film based on a copolymer of lipoic acid-quaternary ammonium salt derivative and chitosan for its utilization in the preservation of grapes. Food Res. Int..

[9] Zou Y., Sun Y., Shi W., Wan B., Zhang H. (2023). Dual-functional shikonin-loaded quaternized chitosan/polycaprolactone nanofibrous film with pH-sensing for active and intelligent food packaging. Food Chem..

[10] Liu M., Chen H., Pan F., Wu X., Zhang Y., Fang X., Li X., Tian W., Peng W. (2024). Propolis ethanol extract functionalized chitosan/Tenebrio molitor larvae protein film for sustainable active food packaging. Carbohydr. Polym..

[11] Rollini M., Mascheroni E., Capretti G., Coma V., Musatti A., Piergiovanni L. (2017). Propolis and chitosan as antimicrobial and polyphenols retainer for the development of paper based active packaging materials. Food Packag. Shelf Life.

[12] Suriyatem R., Auras R., Rachtanapun C., Rachtanapun P. (2018). Biodegradable Rice Starch/Carboxymethyl Chitosan Films with Added Propolis Extract for Potential Use as Active Food Packaging. Polymers.

[13] Siripatrawan U., Vitchayakitti W. (2016). Improving functional properties of chitosan films as active food packaging by incorporating with propolis. Food Hydrocoll..

[14] Yong H., Liu J. (2021). Active packaging films and edible coatings based on polyphenol-rich propolis extract: A review. Compr. Rev. Food Sci. Food Saf..

[15] Abdollahi M., Rezaei M., Farzi G. (2012). Improvement of active chitosan film properties with rosemary essential oil for food packaging. Int. J. Food Sci. Technol..

[16] Gaba A.B.M., Hassan M.A., Abd EL-Tawab A.A., Abdelmonem M.A., Morsy M.K. (2022). Protective impact of chitosan film loaded oregano and thyme essential oil on the microbial profile and quality attributes of beef meat. Antibiotics.

[17] Vishnu Priya N., Vinitha U.G., Meenakshi Sundaram M. (2021). Preparation of chitosan-based antimicrobial active food packaging film incorporated with Plectranthus amboinicus essential oil. Biocatal. Agric. Biotechnol..

[18] Chen S., Wu M., Wang C., Yan S., Lu P., Wang S. (2020). Developed chitosan/oregano essential oil biocomposite packaging film enhanced by cellulose nanofibril. Polymers.

[19] da Costa R.C., Daitx T.S., Mauler R.S., da Silva N.M., Miotto M., Crespo J.S., Carli L.N. (2020). Poly(hydroxybutyrate-co-hydroxyvalerate)-based nanocomposites for antimicrobial active food packaging containing oregano essential oil. Food Packag. Shelf Life.

[20] Kwon S.J., Chang Y., Han J. (2017). Oregano essential oil-based natural antimicrobial packaging film to inactivate Salmonella enterica and yeasts/molds in the atmosphere surrounding cherry tomatoes. Food Microbiol..

[21] Lian H., Shi J., Zhang X., Peng Y. (2020). Effect of the added polysaccharide on the release of thyme essential oil and structure properties of chitosan based film. Food Packag. Shelf Life.

[22] Wang J., Chen C., Xie J. (2022). Loading oregano essential oil into microporous starch to develop starch/polyvinyl alcohol slow-release film towards sustainable active packaging for sea bass (Lateolabrax japonicus). Ind. Crops Prod..

[23] Zhou Y., Sun S., Bei W., Zahi M.R., Yuan Q., Liang H. (2018). Preparation and antimicrobial activity of oregano essential oil Pickering emulsion stabilized by cellulose nanocrystals. Int. J. Biol. Macromol..

[24] Fan S., Wang D., Wen X., Li X., Fang F., Richel A., Xiao N., Fauconnier M.L., Hou C., Zhang D. (2023). Incorporation of cinnamon essential oil-loaded Pickering emulsion for improving antimicrobial properties and control release of chitosan/gelatin films. Food Hydrocoll..

[25] Liu Q.R., Wang W., Qi J., Huang Q., Xiao J. (2019). Oregano essential oil loaded soybean polysaccharide films: Effect of Pickering type immobilization on physical and antimicrobial properties. Food Hydrocoll..

[26] Fujisawa S., Togawa E., Kuroda K. (2017). Nanocellulose-stabilized Pickering emulsions and their applications. Sci. Technol. Adv. Mater..

[27] Ji C., Wang Y. (2023). Nanocellulose-stabilized Pickering emulsions: Fabrication, stabilization, and food applications. Adv. Colloid Interface Sci..

[28] Ni Y., Wu J., Jiang Y., Li J., Fan L., Huang S. (2022). High-internal-phase pickering emulsions stabilized by ultrasound-induced nanocellulose hydrogels. Food Hydrocoll..

[29] Qin X., Li B., Li L., Wang F., Jia S., Xie Y., Zhong C. (2023). Cinnamon essential oil Pickering emulsions: Stabilization by bacterial cellulose nanofibrils and applications for active packaging films. Food Biosci..

[30] Gulzar S., Tagrida M., Prodpran T., Benjakul S. (2022). Antimicrobial film based on polylactic acid coated with gelatin/chitosan nanofibers containing nisin extends the shelf life of Asian seabass slices. Food Packag. Shelf Life.

[31] Imran M., Klouj A., Revol-Junelles A.M., Desobry S. (2014). Controlled release of nisin from HPMC, sodium caseinate, poly-lactic acid and chitosan for active packaging applications. J. Food Eng..

[32] Wang H., Liu H., Chu C., She Y., Jiang S., Zhai L., Jiang S., Li X. (2015). Diffusion and antibacterial properties of nisin-loaded chitosan/poly (L-lactic acid) towards development of active food packaging film. Food Bioprocess Technol..

[33] Cai F., Duan Z., Yu D., Song Z., Lu P. (2025). Antimicrobial packaging activity enhancement by lemon essential oil pickering emulsion stabilized with nanocellulose microgel particles. Food Packag. Shelf Life.

[34] Du Y., Zhang S., Sheng L., Ma H., Xu F., Waterhouse G.I.N., Sun-Waterhouse D., Wu P. (2023). Food packaging films based on ionically crosslinked konjac glucomannan incorporating zein-pectin nanoparticle-stabilized corn germ oil-oregano oil Pickering emulsion. Food Chem..

[35] Liu L., Swift S., Tollemache C., Perera J., Kilmartin P.A. (2022). Antimicrobial and antioxidant AIE chitosan-based films incorporating a Pickering emulsion of lemon myrtle (*Backhousia citriodora*) essential oil. Food Hydrocoll..

[36] Roy S., Rhim J.W. (2021). Gelatin/agar-based functional film integrated with Pickering emulsion of clove essential oil stabilized with nanocellulose for active packaging applications. Colloids Surf. A Physicochem. Eng. Asp..

[37] Tan J.M., Cui R., Hu T.G., Li X.X., Wu H. (2025). Preparation of chitosan/shellac nanoparticles-essential oil Pickering emulsion active film and its application in bread preservation. Food Packag. Shelf Life.

[38] Wang D., Liu L., Chen H., Chi H., Xiang W., Zhang Q., Tang J., Zhang X. (2024). Fabrication and characterization of Zanthoxylum schinifolium essential oil Pickering emulsion stabilized by bacterial cellulose nanofibrils/whey protein isolate complexes and fortified with cinnamaldehyde. LWT.

[39] Yang W., Zhang S., Hu Y., Fu Q., Cheng X., Li Y., Wu P., Li H., Ai S. (2024). Pectin-based film activated with carboxylated cellulose nanocrystals-stabilized oregano essential oil Pickering emulsion. Food Hydrocoll..

[40] Cai L., Yang J., Wang Y., Hu F., Liang B., Qiu W., Song S., Yu L. (2026). Quaternary ammonium chitosan-based film reinforced with antibacterial Pickering emulsion synergistically stabilised by shellac nanoparticles and ε-polylysine for fruit preservation. Food Packag. Shelf Life.

[41] (2024). Standard Test Methods for Gravimetric Determination of Water Vapor Transmission Rate of Materials.

[42] (2024). Standard Test Method for Oxygen Gas Transmission Rate Through Plastic Film and Sheeting Using a Coulometric Sensor.

[43] Li W., Li W., Wan Y., Zhou T., Wang L. (2023). Thymol-loaded Zein-pectin composite nanoparticles as stabilizer to fabricate Pickering emulsion of star anise essential oil for improved stability and antimicrobial activity. J. Food Sci..

[44] Almasi H., Azizi S., Amjadi S. (2020). Development and characterization of pectin films activated by nanoemulsion and Pickering emulsion stabilized marjoram (*Origanum majorana* L.) essential oil. Food Hydrocoll..

[45] Amanda P., Ismadi I., Ningrum R.S., Nabila S., Prasetyo K.W. (2024). Carrageenan functional film integrated with Pickering emulsion of Oregano Oil Stabilized by Cationic Nanocellulose for Active Packaging. Food Sci. Technol. Int..

[46] Chen S., Wu M., Lu P., Gao L., Yan S., Wang S. (2020). Development of pH indicator and antimicrobial cellulose nanofibre packaging film based on purple sweet potato anthocyanin and oregano essential oil. Int. J. Biol. Macromol..

[47] Souza A.G., Ferreira R.R., Paula L.C., Mitra S.K., Rosa D.S. (2021). Starch-based films enriched with nanocellulose-stabilized Pickering emulsions containing different essential oils for possible applications in food packaging. Food Packag. Shelf Life.

[48] Wu M., Zhou Z., Yang J., Zhang M., Cai F., Lu P. (2021). ZnO nanoparticles stabilized oregano essential oil Pickering emulsion for functional cellulose nanofibrils packaging films with antimicrobial and antioxidant activity. Int. J. Biol. Macromol..

[49] Roy S., Priyadarshi R., Rhim J.W. (2022). Gelatin/agar-based multifunctional film integrated with copper-doped zinc oxide nanoparticles and clove essential oil Pickering emulsion for enhancing the shelf life of pork meat. Food Res. Int..

[50] Hao Y., Kang J., Guo X., Sun M., Li H., Bai H., Cui H., Shi L. (2023). pH-responsive chitosan-based film containing oregano essential oil and black rice bran anthocyanin for preserving pork and monitoring freshness. Food Chem..

[51] Fasihi H., Noshirvani N., Hashemi M., Fazilati M., Salavati H., Coma V. (2019). Antioxidant and antimicrobial properties of carbohydrate-based films enriched with cinnamon essential oil by Pickering emulsion method. Food Packag. Shelf Life.

[52] Xu L., Xu X., Xu Y., Huang M. (2023). Oregano essential oil-doped citric acid modified polyvinyl alcohol bio-active films: Properties, bio-functional performance and active packaging application of chicken breast. Food Packag. Shelf Life.

[53] Wu M., Yang J., Chen S., Lu P., Wang R. (2021). TOCNC-g-PEI nanoparticle encapsulated oregano essential oil for enhancing the antimicrobial activity of cellulose nanofibril packaging films. Carbohydr. Polym..

[54] Wu J., Ge S., Liu H., Wang S., Chen S., Wang J., Li J., Zhang Q. (2014). Properties and antimicrobial activity of silver carp (Hypophthalmichthys molitrix) skin gelatin-chitosan films incorporated with oregano essential oil for fish preservation. Food Packag. Shelf Life.

[55] Chen X., Chen W., Lu X., Mao Y., Luo X., Liu G., Zhu L., Zhang Y. (2021). Effect of chitosan coating incorporated with oregano or cinnamon essential oil on the bacterial diversity and shelf life of roast duck in modified atmosphere packaging. Food Res. Int..

[56] Chen X., Hu X., Zhang J. (2024). Properties of an active film based on chitosan/silk fibroin loaded with an essential oil microemulsion and its application in preservation of strawberries. Food Packag. Shelf Life.

[57] Fan X., Yin L., Zhu J., Sun P., Zhu Y., Chen Q., Kong B., Liu Q., Wang H. (2024). The improvement of storage quality of Harbin red sausage by coating with oregano essential oil loaded zein-pectin-chitosan nanoparticles. Food Packag. Shelf Life.

[58] Hernandez-Hosaka C., Park B.R., Kim H., Jung J. (2025). Cellulose-based pickering emulsion films incorporating oregano essential Oil: Valorization of spent coffee grounds for active food packaging. LWT.

[59] Liu Z., Wang H., Yi J., Ma W., Liu X., Chen X., Cao Y., Li L. (2024). Pickering emulsion edible film loaded with oregano essential oil and application in muffin preservation. Packag. Technol. Sci..

[60] Yang L., Liu X., Xiao D., Dong A. (2024). Sustained release composite antimicrobial film containing oregano essential oil microcapsule for postharvest strawberry preservation. Food Packag. Shelf Life.

